# Structural equation model of coping and life satisfaction of community-dwelling older people during the COVID-19 pandemic

**DOI:** 10.1186/s41687-023-00583-x

**Published:** 2023-05-17

**Authors:** Nasreen Lalani, Xu Dongjuan, Yun Cai, Greg W. Arling

**Affiliations:** 1 College of Health and Human Sciences, Purdue University, West Lafayette, IN USA; 2 Center of Aging and Life Course, Purdue University, West Lafayette, IN USA

**Keywords:** Older adults, Coping, Life satisfaction, Optimism, Close relationships, Sense of mastery, Frailty

## Abstract

**Background:**

COVID-19 put older individuals at high risk for morbidity and mortality, isolation, reduced coping, and lower satisfaction with life. Many older adults experienced social isolation, fear, and anxiety. We hypothesized that successful coping with these stressors would maintain or improve satisfaction with life, a crucial psychological outcome during the pandemic. Our study investigated relationships between older people’s coping and life satisfaction during the pandemic and their optimism, sense of mastery, closeness with spouse, family, and friends, and vulnerabilities from frailty, comorbid diseases, memory problems, and dependencies in instrumental activities of daily living (IADL).

**Methods:**

The study was based on a special COVID-19 sample of 1351 community-dwelling older adults who participated in the 2020 Health and Retirement Survey. A comprehensive structural equation modeling was used to test direct and indirect effects, with life satisfaction as the main outcome and coping as a mediator between the other variables and coping.

**Results:**

Most survey respondents were female and between the ages of 65–74 years. They averaged 1.7 chronic conditions, one in seven was frail, about one-third rated their memory as fair or poor, and about one in seven reported one or more difficulties in IADL. As hypothesized—older people with increased sense of mastery and optimism were better able to cope and had greater life satisfaction. In addition, close relationships with friends and with other family members besides the spouse/partner or children contributed to more successful coping, while the interpersonal closeness of all types contributed directly to greater life satisfaction. Finally, older people with more IADL limitations reported greater difficulty coping and lower life satisfaction, and those older people who were frail or had multiple comorbid diseases reported lower life satisfaction.

**Conclusions:**

Optimism, sense of mastery and closeness with family/friends promotes coping and life satisfaction, whereas frailty and comorbidities make coping more challenging and lead to lower life satisfaction particularly during a pandemic. Our study improves on prior research because of its nationally representative sample and formal specification and testing of a comprehensive theoretical framework.

**Supplementary Information:**

The online version contains supplementary material available at 10.1186/s41687-023-00583-x.

## Background

During the COVID-19 pandemic, older people were at high risk for increased morbidity and mortality due to their advanced age, multiple chronic conditions and disability [1, 2]. The majority of older adults experienced increased social isolation, stigmatization, lack of social support, and reduced communications [3, 4]. They reported difficulties in meeting their personal, domestic, or social needs due to lack of care rendered by caregivers and non-availability of social support or formal care services [2]. These stressors posed a challenge to their quality of life and wellbeing which often necessitated psychological adjustment. Prior work on psychological adjustment to COVID-19 has drawn on concepts from a resilience framework [5]. Older adults tend to use their external and internal resources to cope with adversities and can manage significant levels of stress by using their inner resources such as personal capabilities and meaning making; external resources such as social support and networks available in the environment [6–8]. Greater resilience in coping with stress is associated with personality traits such as optimism [9], sense of mastery [10, 11] and being able to draw on support from family and friends [12]. Another study found a positive influence of psychological resilience on older adults’ life satisfaction and improved quality of life [13]. However, we are not certain if this theoretical framework of resilience that has been applied primarily to stress associated with normal life can explain coping and life satisfaction among older people in the context of a pandemic when daily life is disrupted, health is threatened, and social relationships are strained. The purpose of this paper is to examine the role of personality traits, such as optimism and sense of mastery, and social support in the form of relationships with family and friends in promoting the coping and life satisfaction of older adults during a pandemic.

Several studies have been conducted into resilience among community-dwelling older people during the COVID-19 pandemic including factors affecting their coping and life satisfaction. Studies in the US and other countries found that social isolation, fear, and lack of social support during the pandemic negatively affected the coping abilities of older individuals and resulted in poor physical and mental health and wellbeing [4, 14–18]. Other studies found that older individuals who were resilient as evidenced by their positive outlook towards life and those who had adequate social support were better able to cope with daily stressors and adverse life situations [9, 12, 15, 19]. The personality traits such as optimism and sense of mastery helped older people to remain strong and positive, maintain good relationships with their family and friends, enhanced coping and life satisfaction [20–22]. A handful of qualitative studies also suggested that during the pandemic, older people who demonstrated positive coping behaviors and those who were well connected with their family and friends were better able to maintain their daily routines and found to be more satisfied with their lives [23–25]. Findings from these studies are limited due to small-scale or unrepresentative samples, and a relatively narrow set of explanatory variables. Many studies failed to consider the effects of vulnerability due to health conditions and functional impairments, or differences in the importance of social connections between older people and their spouses, adult children, other relatives, and friends.

**Fig. 1:**
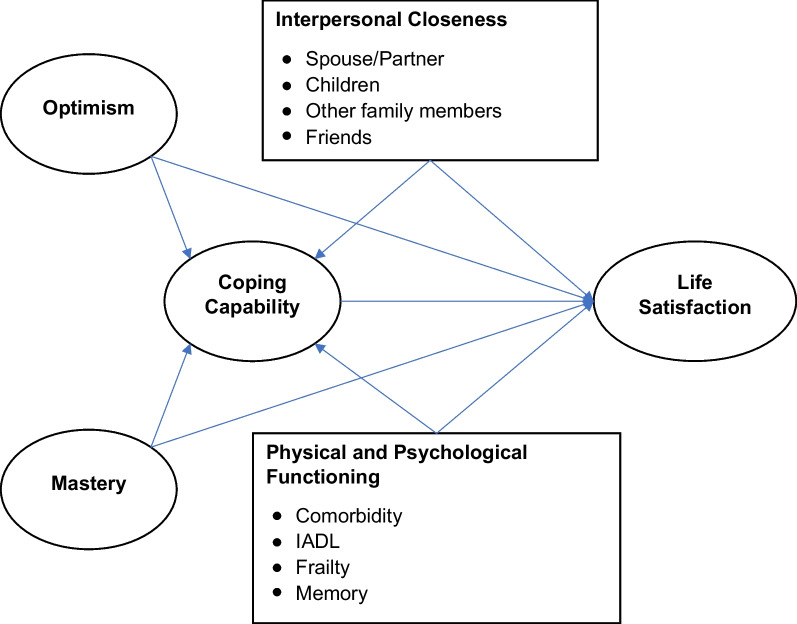
Conceptual framework

In our study, we specify and test a comprehensive theoretical model of coping and life satisfaction during the COVID-19 pandemic among a large national sample of community-dwelling older adults residing in United States (US). Our conceptual framework (Fig. 1) includes personality characteristics (optimism and sense of mastery), interpersonal closeness (with spouse, adult children, other family members, and friends), physical and psychological functioning (frailty, cognitive impairment, limitations in activities of daily living, and comorbidities), along with success in coping with the pandemic (having positive experiences) and satisfaction with life. We apply structural equation modeling (SEM) to investigate these hypotheses:

- *Hypothesis 1* Older adults who are more optimistic, have a better sense of mastery, are closer to their family members and friends, and have better physical and psychological functioning, are more likely to successfully cope with the pandemic and have higher levels of life satisfaction.
- *Hypothesis 2* Older adults with better coping strategies are more likely to have higher levels of life satisfaction during the pandemic.
- *Hypothesis 3* Optimism, sense of mastery, closeness to family members and friends, and better physical and psychological functioning will affect life satisfaction both directly and indirectly when mediated through their effects on coping.

Our study improves on prior research using a large national sample from the Health and Retirement study, its comprehensive theoretical framework, and the formal specification and testing of this framework through a structural equation model.

## Methods

### HRS survey

Data for this study was obtained from the 2020 survey of the Health and Retirement Study (HRS). The HRS consists of longitudinal surveys performed periodically with nationally representative samples of older people in the USA [26, 27]. A special COVID-19 section was incorporated in the 2020 HRS Core interview conducted during March 2020 through June 2021 [26, 27]. The leave-behind, self-administered questionnaire asked a variety of questions about psychosocial topics, e.g., well-being, lifestyle, social relationships, self-related beliefs, etc. COVID-19-related questions were added concerning the impact of the pandemic on participants’ social contacts, activities, feelings, and well-being.

### Sample

A total of 1890 older people were sent the questionnaire by mail followed by a core interview. Of this sample, 539 participants had missing data on all or nearly all items used in the study. The analysis sample for the study consisted of 1351 respondents; 1064 (78.8%) had complete data for all variables; 213 (15.8%) had missing data for one variable, 54 (4.0%) had missing data for 2 variables, and 20 (1.5%) had missing data for 3 variables. We dealt with missing data with procedures described below in the analysis section.

### Measures

#### Outcome: life satisfaction

The latent variable *life satisfaction* was based on items from Diener’s Satisfaction with Life scale, a well-established measure of self-evaluated life quality [28–30]. The measure included five items: “In most ways my life is close to ideal”, “The conditions of my life are excellent”, “I am satisfied with my life”, “So far, I have gotten the important things I want in life”, and “If I could live my life again, I would change almost nothing”. The 7-point response options were: 7 (*strongly agree*), 6 (*somewhat agree*), 5 (*slightly agree*), 4 (*neither agree nor disagree*), 3 (*slightly disagree*), 2 (*somewhat disagree*), and 1 (*strongly disagree*). The higher score indicates a higher level of life satisfaction. The particular scale has shown good construct validity and reliability in other studies also [31–33]. In this study, the Cronbach’s α was 0.87.

#### Mediator: coping ability

The latent variable *coping ability* during the pandemic was assessed by five items designed specifically for the supplemental pandemic survey: since the coronavirus pandemic, “I have learned some positive things from this situation about myself”, “I found greater meaning in work or my other activities and hobbies”, “I now feel more in touch with people in my local community”, “I found new ways to connect socially with other people”, “I am now more appreciative of things that I had taken for granted before”. These items reflect participants’ ability to gain and/or maintain a positive outlook regarding social connectedness and meanings of life in the face of the pandemic as a major source of stress. The 6-point response options were: 6 (*strongly agree*), 5 (*somewhat agree*), 4 (*slightly agree*), 3 (*slightly disagree*), 2 (*somewhat disagree*), and 1 (*strongly disagree*). The higher score indicates better coping ability. In this study, the Cronbach’s α was 0.83.

#### Independent variables

##### Personality characteristics

Latent variables *optimism* and *mastery* were measured through items obtained from the scales used in the HRS. *Optimism* was assessed using three items: “I’m always optimistic about my future”, “In uncertain times, I usually expect the best”, and “Overall, I expect more good things to happen to me than bad”. *Mastery* was assessed by five items: “I can do just about anything I really set my mind to”, “When I really want to do something, I usually find a way to succeed at it”, “Whether or not I am able to get what I want is in my own hands”, “What happens to me in the future mostly depends on me”, and “I can do the things that I want to do”. All items had the same response options regarding participants’ levels of agreement or disagreement with each statement, ranging from 6 (*strongly agree*), 5 (*somewhat agree*), 4 (*slightly agree*), 3 (*slightly disagree*), 2 (*somewhat disagree*), to 1 (*strongly disagree*). The higher score indicates a higher level of optimism or sense of mastery. Both scales had good construct validity and reliability [34–37]. In this study, the Cronbach’s α was 0.81 and 0.91 for optimism and mastery, respectively.

##### Interpersonal closeness

Observed variables for interpersonal closeness were based on the self-rated closeness of ties with a spouse/partner, living children, other family members, and friends. Closeness with spouse/partner was assessed by one item: “How close is your relationship with your partner or spouse?”. The response options ranged from 5 (*very close*), 4 (*quite close*), 3 (*not very close*), 2 (*not at all close*), to 1 (*no spouse/partner*). Closeness with children, other family members, and friends was operationalized as the count of persons they felt had a close relationship with for each relationship. For closeness with children, for example, the responses were categorized into 0 (*no children or no children close*), 1 (*close with one child*), 2 (*close with two children*), 3 (*close with three children*), 4 (*close with four children*), and 5 (*close with five or more children*).

##### Physical and psychological functioning

Physical and psychological functioning was assessed from four domains: comorbidity, instrumental activities of daily living (IADL) difficulty, frailty, and memory status. *Comorbidity* was measured by the count of six health conditions: (1) high blood pressure or hypertension, (2) diabetes or high blood sugar, (3) cancer or a malignant tumor of any kind except skin cancer, (4) chronic lung disease except asthma such as chronic bronchitis or emphysema, (5) heart attack, coronary heart disease, angina, congestive heart failure, or other heart problems, and (6) stroke or transient ischemic attack.

*IADL difficulty* was measured by the count of domains with any difficulty: (1) preparing a hot meal, (2) shopping for groceries, (3) making telephone calls, (4) taking medications, and (5) managing your money, such as paying your bills and keeping track of expenses.

*Frailty* was measured by the Paulson-Lichtenberg Frailty Index (PLFI) including five symptoms: wasting, weakness, slowness, fatigue, and falls [38]. Adapted for the HRS data, *wasting* was identified if a respondent reported a loss of at least 10% of body weight over a 2-year period. *Weakness* was identified if a respondent endorsed the question, “Because of health problems, do you have any difficulty with lifting or carrying weights over 10 pounds, like a heavy bag of groceries?”. *Slowness* was met if a respondent answered “yes” to the question: “Because of a health problem, do you have any difficulty with getting up from a chair after sitting for long periods?”. *Fatigue* was identified if a respondent endorsed the question, “Since we last talked with you in [the last wave], have you had any of the following persistent or troublesome problems: severe fatigue or exhaustion?”. And finally, *falls* were met if a respondent endorsed the question, “Have you fallen down in the past 2 years?”. Individuals who have at least three of the symptoms were identified as frail.

*Memory status* was assessed by one item: “How would you rate your memory at present time? Would you say it is excellent, very good, good, fair, or poor?”.

#### Control variables

Age, gender, and race were all treated as observed variables. There were 3 age groups: 65–74 years, 75–84 years, and 85 + years old. Race was grouped into 2 categories: white and non-white.

### Analysis

The conceptual framework shown in Fig. 1 was tested using structural equation modeling. The modeling took place in two stages consisting of measurement models obtained through confirmatory factor analysis and structural equations containing latent variables from the measurement models and observed variables. The measurement models for the latent variables had good fit to the data, as described in the Additional file 1: Fig. S1–S4.

**Fig. 2:**
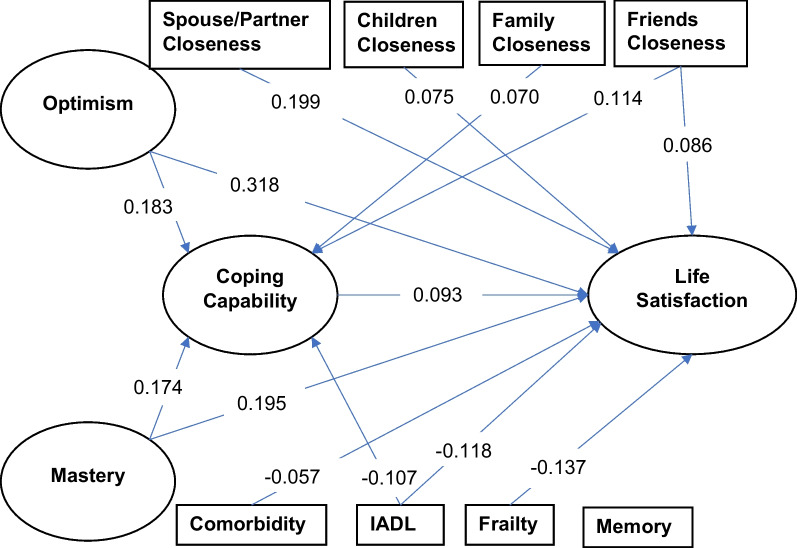
The mediation role of coping on life satisfaction from SEM (n = 1351). *Notes* only show standardized coefficients with *p* < 0.05. IADL: instrumental activities of daily living. SEM: structural equation model

The conceptual model (Fig. 1) consisted of exogenous latent variables of optimism and mastery; exogenous observed variables for interpersonal closeness with spouse/partner, living children, other family members, and friends; and exogenous observed variables for comorbidities, frailty, IADL limitations, memory difficulties. These exogenous variables were hypothesized to have direct effects on the latent variables coping and life satisfaction, as well as indirect effects on life satisfaction which were mediated through coping. Coping was hypothesized to have a direct effect on life satisfaction. To estimate the magnitude of mediating effect, using optimism as an example, we calculated the *indirect effect* of optimism on life satisfaction (i.e., the effect mediated through coping) by multiplying the effect of coping on life satisfaction and the effect of optimism on coping (Fig. 2). The *total effect* was then computed as the sum of the direct effect and the indirect effect of optimism on life satisfaction. In order to adequately estimate parameters and handle missing data, the SEM relied on maximum likelihood estimation with missing values using MLMV (Maximum likelihood with missing values) [39]. MLMV aims to retrieve as much information as possible from observations containing missing values.

As the likelihood ratio chi-square is usually significant with large samples [40], several other fit indices were examined to assess model fit including the Root Mean Square Error of Approximation (RMSEA), the Bentler Comparative Fit Index (CFI), and the Tucker Lewis Index (TLI). A good fit is defined as the RMSEA value smaller than 0.05 and the CFI and TLI values larger than 0.95. And an acceptable fit is defined as the RMSEA smaller than 0.08 and the CFI and TLI values larger than 0.90. The SEM was conducted using Stata software Version 16.1 (StataCorp, College Station, TX).

## Results

Characteristics of the sample are shown in Table 1. Most participants (50.6%) were between the ages of 65–74 years; participants had a mean of 1.7 chronic conditions (SD 1.2). The majority (59.3%) was female, 79.0% white, 14.4% frail, 32.4% with fair to poor self-rated memory, and 13.2% with one or more difficulties in IADL. The interpersonal closeness variables had the following mean scores: spouse/partner 2.8 (SD 1.8), children 2.2 (SD 1.4), family members 2.5 (SD 1.8), friends 2.7 (SD 1.8). The mean scores on the scales with items used in the confirmatory factor analysis were optimism 13.8 (SD 3.4), mastery 23.4 (SD 5.8), coping 20.2 (SD 5.4), and life satisfaction 26.2 (SD 6.9). Means and SDs were based on observations without missing data. The IADL index was the only variable with a coefficient of variation (CV) above 1.00 (SD = 0.71, mean = 0.23). Other CVs were in an acceptable range, particularly for the optimism, mastery, coping and life satisfaction scales.

**Table 1:** Sample characteristics (n = 1351)

	Mean ± SD or N (%)	Range
*Age*		
65–74 years	684 (50.63%)	
75–84 years	500 (37.01%)	
85 + years	167 (12.36%)	
*Gender*		
Male	550 (40.71%)	
Female	801 (59.29%)	
*Race (n = 1349)*		
White	1065 (78.95%)	
Non-white	284 (21.05%)	
Number of chronic conditions (n = 1346)	1.71 ± 1.18	0–6
IADL (n = 1346)	0.23 ± 0.71	0–5
No IADL difficulty	1169 (86.85%)	
Some IADL difficulty	177 (13.15%)	
*Frailty (n = 1297)*		
Yes	187 (14.42%)	
No	1110 (85.58%)	
Self-rated memory (n = 1315)	2.88 ± 0.89	1–5
Excellent	39 (2.97%)	
Very good	267 (20.30%)	
Good	583 (44.33%)	
Fair	354 (26.92%)	
Poor	72 (5.48%)	
*Interpersonal closeness*		
Spouse/partner (n = 1321)	2.75 ± 1.79	1–5
Children (n = 1338)	2.15 ± 1.39	0–5
Other family members (n = 1347)	2.50 ± 1.82	0–5
Friends (n = 1342)	2.74 ± 1.81	0–5
Optimism (n = 1295)	13.80 ± 3.41	3–18
Mastery (n = 1319)	23.42 ± 5.81	5–30
Coping (n = 1258)	20.17 ± 5.43	5–30
Life satisfaction (n = 1309)	26.15 ± 6.89	5–35

*SD* Standard deviation, *IADL* Instrumental activities of daily living

(Please note Table 1: Sample Characteristics should be placed here).

Results of the structural equation modeling are shown in Table 2 and Fig. 2. The full structural equation model is presented in the Additional file 1: Fig. S5. The model provided a good fit to the data: RMSEA = 0.042, CFI = 0.943, and TLI = 0.933. Overall, the model predicted approximately 14% of the variance in coping capability (*R*^2^ = 0.139), and approximately 30% of the variance in life satisfaction (*R*^2^ = 0.303).

**Table 2:** The mediation role of coping on life satisfaction from SEM (n = 1351)

	Coping	Life satisfaction			
	Direct effect	Direct effect	Indirect effect through coping	Total effect	Indirect effect/total effect (%)
*Coping*	–	0.093**	–	–	–
*Personality*					
Optimism	0.183***	0.318***	0.017*	0.335***	5.11
Mastery	0.174***	0.195***	0.016*	0.211***	7.71
*Interpersonal closeness*					
Spouse/partner	− 0.041	0.199***	− 0.004	0.195***	–
Children	− 0.018	0.075**	− 0.002	0.073**	–
Other family members	0.070*	0.016	0.007	0.023	–
Friends	0.114***	0.086**	0.011*	0.096***	11.07
*Physical and psychological functioning*					
Comorbidity	0.016	− 0.057*	0.001	− 0.055*	–
IADL	− 0.107**	− 0.118***	− 0.010*	− 0.128***	7.77
Frailty	− 0.0002	− 0.137***	− 0.00001	− 0.137***	–
Memory	0.019	0.052	0.002	0.054	–

All coefficients are standardized

*IADL* Instrumental activities of daily living, *SEM* Structural equation model

*p < 0.05, **p < 0.01, ***p < 0.001

The standardized path coefficients including direct and indirect effects are provided in Table 2. As we hypothesized, closeness with friends (β = 0.114, *p* < 0.001) and closeness with family members (β = 0.070, *p* < 0.050) had significant positive effects on coping during the pandemic. However, closeness with spouse/partner and with living children were not significantly related. Self-rated optimism (β = 0.183, *p* < 0.001) and mastery (β = 0.174, *p* < 0.001), also had significant positive effects on coping. Among the physical and psychological functioning variables, IADL difficulties had a significant negative effect on coping (β =  − 0.107, *p* < 0.010).

In line with our hypotheses, optimism (β = 0.318, *p* < 0.001), mastery (β = 0.195, *p* < 0.001) closeness with spouse/partner (β = 0.199, *p* < 0.001), closeness with children (β = 0.075, *p* < 0.010), and closeness with friends (β = 0.086, *p* < 0.010) all had significant positive direct effects on life satisfaction. On the other hand, frailty (β =  − 0.137, *p* < 0.001), multiple comorbidities (β =  − 0.057, *p* < 0.050), and greater IADL limitations (β =  − 0.118, *p* < 0.001) had negative direct effects on life satisfaction. As we hypothesized a higher coping score (β = 093, *p* < 0.010) had a positive direct effect on life satisfaction.

Four variables had relatively small yet significant indirect effects on life satisfaction that were mediated through the coping variable. Closeness with friends (β = 0.011, *p* < 0.05), optimism (β = 0.017, *p* < 0.05), and mastery (β = 0.016, *p* < 0.05) had positive indirect effects, while IADL limitations had a negative indirect effect (β =  − 0.010, *p* < . 05). Hence, we found that coping mediated the relationships between the four variables and life satisfaction accounting for 5.1–11.1% of the total effect (optimism: 5.1%, mastery: 7.7%, IADL limitations: 7.8%, and closeness with friends: 11.1%).

## Discussion

Our hypotheses were largely supported in our study testing a structural equation model of coping and life satisfaction using a large national sample of older people in the US during the COVID-19 pandemic. More successful coping contributed to higher life satisfaction. Older people with increased sense of mastery and optimism were better able to cope and had greater life satisfaction. The greater levels of mastery and optimism contributed directly to better life satisfaction and indirectly through its relationship to more successful coping.

Our study lends further support for the influence of key variables in promoting successful coping and higher life satisfaction in the face of stressful disruptions of daily life, threats to health, and strained social relationships. From a psychological perspective, optimism and a greater sense of mastery serve as a defense against various age-related challenges and encourage positive aging adaptation [9, 41, 42]. These psychological reserves allow older individuals to exercise more control over their lives, their physical activities, and daily routines as well as provide them with a greater sense of hope for the future [9, 41, 42].

We also found support for the hypothesis that close personal relationships enhanced coping and contributed to greater life satisfaction among older adults during the pandemic. These findings are in line with prior research during the pandemic which found that having close relationships with spouse, family and friends enhanced social support among older people, and increased their likelihood of positive coping and wellbeing [12, 18, 43, 44]. Notably, our study was unique because we modeled several types of interpersonal closeness separately to determine if closeness to spouse, children, other family and friends might affect coping or life satisfaction differently. Close relationships with friends and with other family members besides the spouse/partner or children contributed to more successful coping. Interestingly, close relationships with a spouse/partner and adult child were not significantly related to coping. On the other hand, we also found that interpersonal closeness of all types contributed directly to greater life satisfaction during the pandemic. Closeness to a spouse/partner and to friends made the greatest contribution (strongest standardized effect), although closeness to children and other family members also contributed. Relationships with friends had an indirect contribution: older people with closer friends were better able to cope better and had greater life satisfaction. Additional research is necessary to understand why types of interpersonal relationships differed in their effect on coping and life satisfaction. Caregiving responsibilities could play a role in these relationships, particularly among frail or functionally dependent older people. The burden placed on spouse or adult children who are most likely to be primary caregivers may be heightened during the pandemic causing strained relationships.

We found that older people with more IADL limitations had greater difficulty coping and lower life satisfaction. Frailty and multiple comorbid diseases also contributed to lower life satisfaction. Poor health and functional limitations can lower satisfaction with life during “normal” times; this effect was likely exacerbated by restrictions during the pandemic and the greater vulnerability to serious symptoms or death. Similar findings were reported in other studies during the pandemic [45, 46]. However, having psychological and social resources might buffer the effect of frailty on life satisfaction [47]. These findings point to the complexity of how older people and their caregivers respond to frailty and functional dependency.

## Strengths and limitations

Our study has several strengths. Our study contain data from a large national survey of community-dwelling older adults during the COVID-19 pandemic. The measure of coping was based on items specific to the pandemic. Other important domains, such as optimism, mastery, and life satisfaction were measured with items drawn from established scales. We specified and tested formal hypotheses with structural equations models which estimated direct and indirect effects with simultaneous statistical controls for all variables in the models. Our findings were generally consistent with our hypotheses.

Limitations of the study should also be noted. The mail-out HRS supplemental survey had a high rate of non-responses for about 30% of respondents who we excluded from the analysis. The cross-sectional survey used in the study limits our ability to infer any causality among the relationships studied. Our specification of the conceptual model is only one of several possible sets of relationships between constructs. Taking another perspective, close relationships could be viewed as endogenous variables, where older people who are more optimistic about life would be better able to develop close relationships with their family and friends and be more satisfied with life [9, 12]. Finally, although we achieved an acceptable fit to the data, some significant coefficients in our model were weak and the model explained a relatively small amount of variance in coping and life satisfaction. Unmeasured variables could have affected these outcomes in the study.

## Conclusions and implications

Our study highlights the importance of personality factors such as optimism and mastery, as well as close relationships with spouse/partner, children, other family and friends in older adults’ ability to cope and remain satisfied with life during trying times. The study also points to the vulnerability of older people with limitations in daily activities, comorbid conditions, and frailty. Strategies pursued by wellness programs or other community interventions for older people should seek to maintain or enhance feelings of optimism and personal mastery, close interpersonal ties, and informal support, especially during difficult times. These efforts should be targeted especially at older people in poor health and with physical or functional disabilities.

## Supplementary Information

**Additional file 1: Fig. S1.** Confirmatory factor analysis for optimism latent variable.** Fig. S2.** Confirmatory factor analysis for mastery latent variable.** Fig. S3.** Confirmatory factor analysis for coping capacity latent variable.** Fig. S4.** Confirmatory factor analysis for life satisfaction latent variable.** Fig. S5.** The full SEM.

## Data Availability

The data, analytic methods or materials are available to other researchers for replication purposes. Data for this study came from the 2020 Health and Retirement Study (HRS) COVID-19 project incorporated in the 2020 HRS Core interview, conducted from March 2020 through June 2021 (Health and Retirement Study, 2022). The data can be accessed from https://hrs.isr.umich.edu/about. HRS/Health and Retirement Study.
