# Supplementary material for: Structural equation model of coping and life satisfaction of community-dwelling older people during the COVID-19 pandemic
Source: J Patient Rep Outcomes. 2023 May 17;7:46. doi: 10.1186/s41687-023-00583-x (PMC10191072; doi:10.1186/s41687-023-00583-x)
Supplement: Supplementary file 1 — Additional file 1: Fig. S1. Confirmatory factor analysis for optimism latent variable. Fig. S2. Confirmatory factor analysis for mastery latent variable. Fig. S3. Confirmatory factor analysis for coping capacity latent variable. Fig. S4. Confirmatory factor analysis for life satisfaction latent variable. Fig. S5. The full SEM. [file 41687_2023_583_MOESM1_ESM.docx]

**Supplemental Materials**

**Figure 1. Confirmatory factor analysis for *optimism* latent variable**

be optimistic about my future

expect the best

expect more good things to happen

Ꜫ_1_

Ꜫ_2_

Ꜫ_3_

0.70^***^

0.86^***^

0.75^***^

Notes: Standardized coefficients presented. ***p<0.001. CFI = 1.00, TLI = 1.00, and RMSEA < 0.01.

**Figure 2. Confirmatory factor analysis for *mastery* latent variable**

can do just about anything

find a way to succeed

able to get what I want

Ꜫ_1_

Ꜫ_2_

Ꜫ_3_

0.81^***^

0.80^***^

future mostly depends on me

can do the things that I want to do

Ꜫ_4_

Ꜫ_5_

0.78^***^

0.69^***^

0.85^***^

Notes: Standardized coefficients presented. ***p<0.001. CFI = 0.997, TLI = 0.974, and RMSEA = 0.09 (95% CI: 0.05,0.14).

**Figure 3. Confirmatory factor analysis for *coping capacity* latent variable**

have learned some positive things

found greater meaning

feel more in touch with people

Ꜫ_1_

Ꜫ_2_

Ꜫ_3_

0.75^***^

0.81^***^

found new ways to connect socially

more appreciative of things

Ꜫ_4_

Ꜫ_5_

0.58^***^

0.59^***^

0.60^***^

Notes: Standardized coefficients presented. ***p<0.001. CFI = 0.998, TLI = 0.977, and RMSEA = 0.06 (95% CI: 0.02,0.12).

**Figure 4. Confirmatory factor analysis for *life satisfaction* latent variable**

my life is close to ideal

conditions of life are excellent

satisfied with my life

Ꜫ_1_

Ꜫ_2_

Ꜫ_3_

0.79^***^

0.80^***^

have gotten the important things

would change almost nothing

Ꜫ_4_

Ꜫ_5_

0.84^***^

0.70^***^

0.55^***^

Notes: Standardized coefficients presented. ***p<0.001. CFI = 1.00, TLI = 1.00, and RMSEA < 0.01.

**Figure 5. The full SEM**

**Spouse/Partner** **Closeness**

**Children Closeness**

**Family Closeness**

**Friends Closeness**

**Comorbidity**

**IADL**

**Frailty**

**Memory**

0.183^***^

0.174^***^

0.195^***^

0.318^***^

0.093^**^

0.114^***^

0.086^**^

0.199^***^

0.075^**^

-0.118^***^

**Age 75-84**

**Age 85+**

**Female**

**White**

0.070^*^

-0.107^**^

-0.057^*^

0.016

-0.041

-0.018

-0.0002

0.052

0.019

0.105^*^

0.062

-0.112^**^

0.101^**^

-0.061

-0.019

0.008

0.016

-0.137^***^

0.042

Notes: Standardized coefficients presented. *p<0.05 **p<0.01 ***p<0.001. IADL: instrumental activities of daily living. SEM: structural equation model
